# *Agrilus mali* Matsumara (Coleoptera: Buprestidae), a new invasive pest of wild apple in western China: DNA barcoding and life cycle

**DOI:** 10.1002/ece3.4804

**Published:** 2018-12-27

**Authors:** Tohir A. Bozorov, Zhaohui Luo, Xiaoshuang Li, Daoyuan Zhang

**Affiliations:** ^1^ Key Lab of Biogeography and Bioresource in Arid Land, Xinjiang Institute of Ecology and Geography Chinese Academy of Sciences Urumqi China; ^2^ Institute of Genetics and Plants Experimental Biology Uzbek Academy of Sciences Tashkent Region Uzbekistan

**Keywords:** *Agrilus mali*, DNA barcode, invasive species, life cycle, phylogeny, wild apple

## Abstract

*Agrilus mali* Matsumara (Coleoptera: Buprestidae) is a wood‐boring beetle distributed to eastern China that occasionally injures apple species. However, this wood‐boring beetle is new to the wild apple forests (*Malus sieversii*) of the Tianshan Mountains (western China) and has caused extensive tree mortality. The development of a biological control program for these wild apple forests is a high priority that requires exploration of the life cycle, DNA barcoding and taxonomic status of *A. mali*. In this study, to determine the diversity of invasive beetles, a fragment of the mitochondrial *cytochrome oxidase* gene was analyzed. Based on the results, beetles from Gongliu and Xinyuan counties of Xinjiang were identical but differed from those in the apple nursery of Gongliu by a single‐nucleotide substitution. We summarize the taxonomic status, relationships, and genetic distances of *A. mali* among other *Agrilus* species using the Tajima‐Nei model in maximum likelihood phylogeny. Analysis revealed that *A. mali* was closely related to *Agrilus mendax* and both belong to the *Sinuatiagrulus* subgenus. The life cycle of *A. mali* was investigated based on a monthly regular inspection in the wild apple forests of Tianshan. Similar to congeneric species, hosts are injured by larvae of *A. mali* feeding on phloem tissue, resulting in serpentine galleries constructed between bark and xylem that prevent nutrient transport and leading to tree mortality. Future studies will focus on plant physiological responses to the invasive beetles and include surveys of natural enemies for a potential classical biological control program.

## 1 INTRODUCTION

Over 3,000 species of the genus *Agrilus* are distributed globally and among them, 686 species‐group taxa injure 941 host–plant species from 308 genera and 84 plant families (Jendek & Poláková, 2014). Among the beetles of the genus, many species are invasive exotics (Jendek & Poláková, 2014; Sydnor, Bumgardner, & Todd, 2007) that affect the economic productivity of timber resources and threaten forest ecosystems (Gandhi & Herms, 2010a; Holmes, Aukema, Holle, Liebhold, & Sills, 2009; Liebhold, Macdonald, Bergdahl, & Maestro, 1995).

The biology of some species is well studied because of their importance in agriculture and forestry, including the most studied and infamous, emerald ash borer (EAB) *Agrilus planipennis *Fairmaire. Emerald ash borer is native to eastern Asia and as a pest of ash trees of *Fraxinus *spp. in North America, has caused extensive tree mortality since first detected in 2002 (Chamorro, Volkovitsh, Poland, Haack, & Lingafelter, 2012; Jendek & Poláková, 2014). *Agrilus planipennis* was likely introduced from eastern China to North America in the 1990s (Siegert et al., 2010), and as of March 2018, EAB has expanded to 21 US states and two provinces of Canada (Emerald Ash Borer Information Network; Herms & McCullough, 2014). Closely related to EAB, the bronze birch borer *Agrilus anxius *Gory, native to North America, damages exotic birch more than native species (Herms, 2002). A third species, *Agrilus sulcicollis* Lacordaire develops in oak trees which are native to most of Europe and was first reported in North America in 2008 (Haack, Petrice, & Zablotny, 2009; Jendek & Grebennikov, 2009). A fourth species, the East Asian buprestid *Agrilus smaragdifrons* Ganglbauer has been reported for the first time in the Western Hemisphere and suggests establishment of this metallic wood‐boring beetle in the northeastern US (Hoebeke et al., 2017).  A fifth species, *Agrilus ribesi* Schaefer is an invasive that has established in North America that is mostly native to the Eurasian continent (Jendek, Grebennikov, & Bocak, 2015). All these species are intercontinental invaders with cross‐continental invasions. However, *Agrilus* species can also invade within a continent or locally. Emerald ash borer has been expanding to European Russia and likely to most of Europe (Orlova‐Bienkowskaja, 2014). Another example is the gold‐spotted oak borer, *Agrilus auroguttatus* Schaeffer, is an invasive oak borer in California, but is native to southern Arizona of the USA (Lopez, Rugman‐Jones, Coleman, Hoddle, & Stouthamer, 2014). The establishment of introduced invasive species has become problematic on a large scale causing very high levels of ecological and economic (Lodge et al., 2016). Most invasive wood‐boring insects cause mortality of trees that results in direct or indirect effects on forest ecosystem processes (Gandhi & Herms, 2010a; Herms & McCullough, 2014). As an example, mortality of ash trees affects several insects and arthropod species that feed on ash trees and may be at risk of co‐extirpation (Gandhi & Herms, 2010b; Herms & McCullough, 2014). Increasing international or domestic exports/imports of timber, firewood, and seedlings without quarantine assessment accelerates the distribution of wood‐boring beetles to non‐native habitats (Haack, Herard, Sun, & Turgeon, 2010; Poland & McCullough, 2006; Sydnor et al., 2007). Among the *Agrilus* species, an invasive *Agrilus mali* Matsumara injures *Malus* species (Matsumura, 1924), including their endangered wild relatives (Ji, Ji, & Huang, 2004).

Nine species among the genus *Agrilus* injure *Malus *species including *M. domestic*, *M. pumila, M. silvestris,* and *M. sieversii* while only *A. mali *Matsumara is known to attack *M. sieversii *(Jendek & Poláková, 2014; Ji et al., 2004). *Agrilus mali* is native to the eastern part of the Eurasian continent, primarily northeastern China and Amurskaya oblast, Khabarovsk and Primorskiy Kray of the Russian Federation and the Korean Peninsula and Japan (Chebanov, 1977; Cui, Liu, & Liu, 2015; Matsumura, 1924; Nikritin, 1994). *Agrilus mali *sporadically (i.e., non‐epidemically) injures *Malus *(*M. domestica and M. pumila*) and *Pyrus *species (Chebanov, 1977; Matsumura, 1924; Nikritin, 1994). The first record of *A. mali* in western China was in 1993 due to the introduction of domesticated apple seedlings from Shangdong province to Xinyuan orchard in Ili Valley, Xinjiang for breeding purpose (Ji et al., 2004). Within 10 years after introduction, the beetle had escaped and rapidly distributed in the wild apple forests of Xinyuan located in the middle of Tianshan Mountains killing thousands of trees. Notably, this beetle only sporadically injures other species of apple tree, in addition to *M. sieversii*, that grow in native habitats of eastern regions of China (Cui et al., 2015); however, the lack of a co‐evolutionary history likely led to the extensive mortality in the wild apple forests of Tianshan.

Forests of the Tianshan Mountains are rich in wild fruit relatives of domesticated species. The forest composition of wild fruits in the Chinese Tianshan (eastern Tianshan) is estimated at 38%, whereas in the central Asian portion, the estimate is 62% (Lin & Lin, 2000). *Malus sieversii* (Ledeb.) Roem. is a wild apple species native to central Asia found in Kazakhstan, Kyrgyzstan, Tajikistan, Uzbekistan, and northeastern Afghanistan, and in western China, the species is widely distributed in the Tianshan sub‐mountain area (Lin & Lin, 2000; Sokolov, Svjazeva, & Kubly, 1980). Although wild apple forests occupy an estimated 93% among the wild fruit forests in Chinese Tianshan, compared with 78% for the central Asian portion (Lin & Lin, 2000), the wild apple forests are not well studied. The wild apple *M. sieversii* is the primary progenitor of all cultivated domesticated apple species (Duan et al., 2017; Harris, Robinson, & Juniper, 2002; Hokanson et al., 1998; Richards et al., 2009; Zhang, Zhang, & Wang, 2015) and therefore is a globally important resource for apple breeding because of the rich genetic diversity. Thus, the ex and in situ preservation of this species is globally important (Hokanson et al., 1998; Yang et al., 2016).

Unfortunately, in the past two decades, *A. mali* has heavily attacked this species, which has caused extremely high mortality in the apple forests of the Tianshan Mountains since the first detection in 1993 (Ji et al., 2004). Since then the estimate is that 40% (3,866.67 hm^2^) of the area of wild apple forest in Tianshan has been damaged, with the death of 666.67 hm^2^ of forest (Wang, 2013). Moreover, the vulnerability of wild apple increases under *A. mali* attack because fungi are also promoted. Among the fungi, Valsa canker, *Valsa mali *var. *mali *(Vmm), has been one of the important threatening factors for the wild apple forests (Wang, Li, et al., 2014a; Wang, Zang, Yin, Kang, & Huang, 2014b). With the attack of wild apple branches by *A. mali*, the vulnerability to *V. mali* invasion increases, which can accelerate tree mortality (Wang, Li, et al., 2014a; Wang, Wei, Huang, & Kang, 2011; Wang, Zang, et al., 2014b). Currently, *A. mali* is the primary threat to wild apple in the Tianshan region of Xinjiang‐Uyghur Autonomous Province and is considered as a quarantine pest. Thus, pest management and biological control on *A. mali* are quite an urgent task in ecological recovery of the Tianshan wild apple forest.

Development of pest management programs that use chemical, biological, or cultural (i.e., pruning of infested branches) control options are essential. However, application of pesticides often does not provide expected results. Treatment of an infested tree may continue canopy decline during the first year and if treatment is effective, the canopy will usually begin to improve in the second year of treatment. Probably as the tree repairs its vasculature system after infestation has been reduced (Herms et al., 2009). Early in the discovery of the EAB invasion in North America, a survey was begun to seek natural enemies, parasitoids, and pathogens that parasitized EAB (Cappaert, McCullough, Poland, & Siegert, 2005; Liu et al., 2007). A classical biological control approach might efficiently eradicate an invasive insect species in a new habitat, or manage providing long‐term, widespread control of the pest without application of chemicals or other strategies that require human intervention (Paine et al., 2015; Van Driesche et al., 2010). For instance, introduced encyrtid egg parasitoid *Avetianella longoi* (Siscaro) from Australia to California resulted in complete biological control of wood borer *Phoracantha semipunctata* throughout the state (Hanks, Gould, Paine, Millar, & Wang, 1995). Currently, several natural enemies have been found that prey on EAB in different life stages, which co‐evolved in Asia with EAB (Duan, Bauer, Abell, Ulyshen, & Driesche, 2015; Gould, Ayer, & Fraser, 2011). Unfortunately, little is known about the natural enemies that could be used to control invasive *A. mali*. Liu et al. (2010) discussed preliminary research on the control of *A. mali* and proposed that *Atanycolus *species could be a dominant natural enemy for these invasive insects. Additionally, the wasp species *Spathius agrili* is proposed as a parasite of both EAB and *A. mali* (Yang, Wang, Gould, & Wu, 2008), and four species of bethylid wasps parasitize *A. mali* larvae (Jiang, Yang, Wang, & Hou, 2015; Wang, Yang, Zhang, Wang, & Tang, 2014c). However, few successful practices had been reported and assessed.

The establishment and behavioral ecology of the invasive wood‐boring insect *A. mali* in the forests of Tianshan must be investigated because of the significance for in situ conservation of wild apple populations in their natural habitat. First, the taxonomic traits and systematics position of the *A. mali* should be made clear. In this study, we explored the diversity among *A. mali* specimens in the forests of the Tianshan Mountains with partial *cytochrome oxidase* (*COI*) sequences using DNA barcoding. The mitochondrial *COI* gene is widely used to separate species that are morphologically indistinguishable in early stages and also in insect inter‐ and intra‐population comparative analyses (Havill, Montgomery, Yu, Shiyake, & Caccone, 2006; Rugman‐Jones, Hoddle, & Stouthamer, 2010; Wilson, 2012). Using maximum likelihood tree analysis following, a phylogenetic analysis determined taxonomic status among *Agrilus* species. Additionally, we report on insect description, the life cycle of the invasive beetles and described the taxonomic traits of *A. mali*. This research provides basic information that is fundamental for initiating the search for natural enemies of *A. mali* in order to use in a classical biological control program.

## 2 MATERIALS AND METHODS

### 2.1 Research site, specimen collection, and DNA extraction

**Figure 1:**
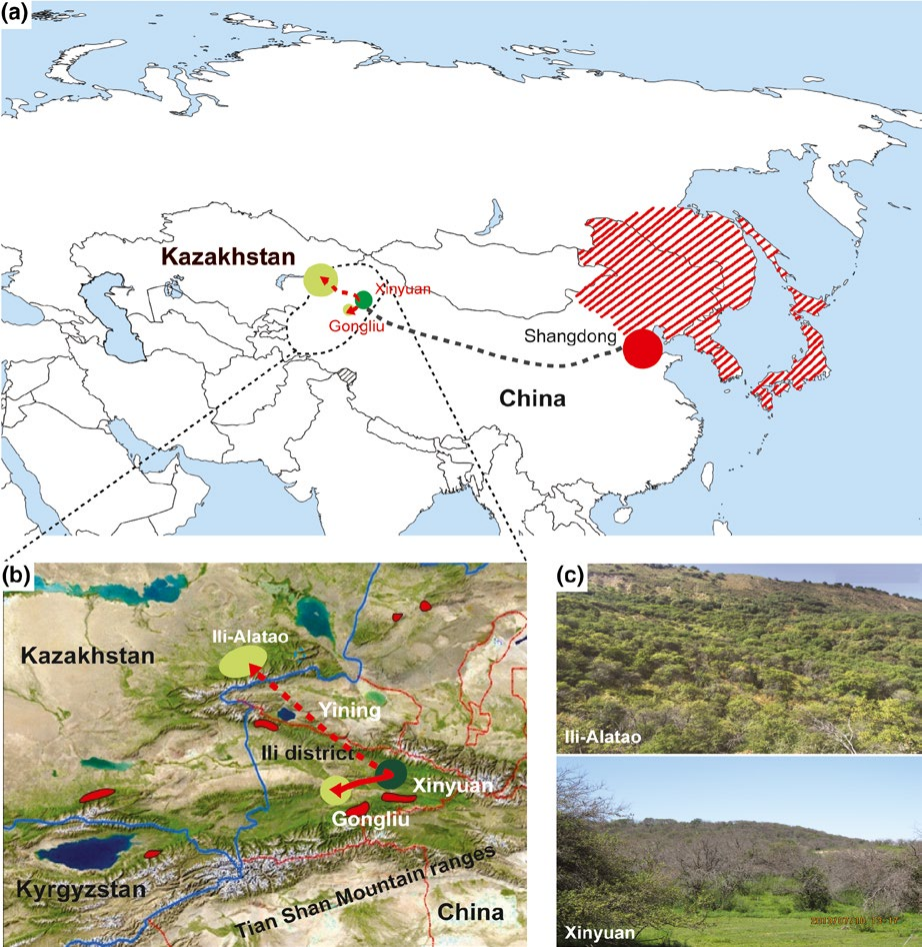
Invasion map of *Agrilus mali* from western to eastern part of China. Distribution of *A. mali *in eastern Asia habitat and the establishment in central Asia (western China) (a). The area with red lines indicates proposed habitats of *A. mali* in eastern Asia. The area with dashed line depicted on the map shows proposed an introduction (black dashed line) of the beetle from the eastern part (red circle) of western China. Dark green circle demonstrates a location where apple population was highly damaged compared to light green circle locations (b). The red line indicates direct introduction of beetle to nearest area, whereas the red dashed line indicates proposed invasion from high damaged area to neighboring country. The area in red with black line indicates locations of wild apple forests. Examples of damaged wild apple forest views in Xinyuan and Ili‐Alatao (c)

The research was conducted in Mohe Village including a nursery (43°34N 83°18W; 43°13N 83°47W; 43°51N 82°15W) of Gongliu County and at the Yili Botanical Research Station (43°22N 83°34W) of Xinyuan County (Ili‐Kazakh District) of Xinjiang‐Uyghur Autonomous Province, China, which are located in the Ili Valley of the Tianshan Mountains (Figure 1). The annual average temperature in the Tianshan Mountains is 6.1°C, with a maximum temperature of 35°C and a minimum temperature of −19°C (Chen, Wei, & Liu, 2013). Among the Rosaceae, wild apple is major species fruit forest, but also a few wild pear species can be found. Insect specimens were collected between 2014 and 2016 from infested wild apple trees in Xinyuan and Gongliu counties, including a wild apple nursery in Gongliu (Table 1, Figure 1). Ten beetle specimens were randomly collected from leaves of infested trees from different sites of forest. All collected specimens were immediately preserved in 95% ethanol and stored at −20°C before use for morphological description and DNA barcoding. Individual *A. mali* adult specimens were removed from tubes and air‐dried on filter paper for homogenization. Beetles were ground under liquid nitrogen with mortar and pestle. Fine‐powdered tissue was transferred to 2 ml Eppendorf tubes. Next, we used a Blood and Tissue kit for DNA isolation (Qiagen, Germany) following the manufacturer's protocols. Following 0.8% agarose (Invitrogen, USA) gel electrophoresis, total DNA was visualized using a Tanon 2500R Gel Documentation System (Tanon Science and Technology, China).

**Table 1:** Collection informations for *Agrilus mali* populations in western Tianshan Mountains of China

Locality	County	Province	GPS	Collection date
Tianshan	Gongliu	Xinjiang‐Uyghur Autonomous	43°34N 83°18 W	02/08/2015
Tianshan	Gongliu	Xinjiang‐Uyghur Autonomous	43°13N 83°47 W	06/07/2016
Tianshan	Gongliu (Farmland)	Xinjiang‐Uyghur Autonomous	43°51N 82°15 W	26/06/2016
Tianshan	Xinyuan	Xinjiang‐Uyghur Autonomous	43°22N 83°34 W	01/07/2014

### 2.2 PCR and sequencing

A degenerative primer pair COIFor‐1 (5′‐GGAAAYCCHGGDGCWTTAATTGG‐3′) and REVCOI‐3 (5′‐TCTCCCCCYCCTGCYGGGTCAAA‐3′) was designed from aligned *COI* sequences from *Agrilus* species to amplify a 530 bp fragment of the *COI* gene of *A. mali*. For designing primers, the most conserved region among the aligned congeneric *COI* sequences was selected. Then, nucleotide mixes were included into the primer sequence to be most complementary to the *COI* region. Amplifications were performed in a total volume of 20 µl containing 10 µl of PrimeSTAR HS (Premix) (Takara, Japan) that contained an appropriate concentration of dNTP and Taq‐polymerase, 1 µl of (0.2 µM of each primer), and 4 µl of DNA template. Amplification was performed on a Veriti thermocycler (Applied Biosystems, USA). PCR conditions were as follow: 5 min at 95°C for the initial step, 35 cycles of 15 s at 94°C for denaturation, 30 s at 52°C for annealing, and 1 min at 72°C for an elongation step, and 5 min at 72°C for final elongation. PCR products were visualized on 1.5% agarose gel. PCR products were ligated into TA cloning vector pMD20‐T (Takara) and then transformed into *Escherichia coli* Trans5ɑ strain (Transgen Biotech, China). Colony PCR was conducted to validate gene insertion using gene‐specific and M13 sequencing primers. Overnight cultured cells were sent to the Beijing Genomics Institute (China) for sequencing. The sequences were trimmed and edited using Bio Edit v7.2.5. *COI* sequences were uploaded to the NCBI (KY982844, KY982844, KY982846, KY982847, KY982848, KY982849, KY982850, KY982851, KY982852).

### 2.3 Phylogenetic analysis

To determine the similarities of *A. mali COI* sequence with other *Agrilus* species, a BLAST search was performed and partial *COI* gene sequences of congeneric species were obtained from GenBank databases (NCBI and BOLD). Thirty‐five *COI* sequences of *Agrilus* species were retrieved from NCBI and BOLD systems using a BLASTn search. Multiple sequence alignment was performed with MEGA7 using the CLUSTALW tool. The bootstrap method with 1,000 replicates and the Van Driesche,‐Nei method in MEGA7 (Felsenstein, 1985; Kumar, Stecher, & Tamura, 2016; Tajima & Nei, 1984) were used for pairwise analysis of sequences. The number of base substitutions per site was analyzed for all sequences. Nucleotide percentage of 1st + 2nd + 3rd codon positions was calculated using MEGA7. All positions containing gaps and missing data were eliminated from the data set. The neighbor‐joining (NJ) (Saitou & Nei, 1987) tree was built using MEGA7 to resolve similarity.

The evolutionary history was inferred by using maximum likelihood method based on Hasegawa–Kishino–Yano (HKY) model with the rapid bootstrap method (500 replicates) with MEGA7. Initial tree(s) for the heuristic search were obtained by applying the BioNJ method to a matrix of pairwise distances estimated using the Maximum Composite Likelihood approach by applying uniform rates. We conducted parallel Bayesian MCMC analysis based on HKY model with BEAST V.1.8.4 (http://beast.community) which was used to run 1,000,000 generations (Drummond, Suchard, Xie, & Rambaut, 2012).

Images of congeneric insects were obtained from insect databases BOLD System (http://www.boldsystems.org), Bug Guide (http://bugguide.net/), and Biodiversity Map TAXA (http://baza.biomap.pl), www.zin.ru/Animalia/Coleoptera/eng/agrprapo.htm, www.flickr.com/photos/eurythyrea, www.coleoptera.org.uk/, www.flickr.com/photos/kohichiroh, www.biolib.cz/en/image/id184987 and Jendek and Grebennikov (2011).

### 2.4 Insect description and life cycle

The format, style of the insect description and morphological terms were followed as described by Jendek and Grebennikov (2011). A binocular stereomicroscope Olympus SZX10 (https://www.olympus-ims.com) was used for morphological description of beetles. Larval instars were distinguished following Wang, Zhang, Yang, and Wang (2013). Briefly, binocular microscope with an eyepiece scale was used to measure peristoma width, urogomphus length, and larval length, and molting rate. Crosby ratio was determined to verify the accuracy in the grouping process of the larval instars (Craig, 1975).

Based on monthly regular inspection of wild apple trees attacked by larvae in forests, the life cycle of *A. mali* was observed from March 2016 through September 2017. Observations were conducted once per month. Briefly, 10 wild apple trees were randomly selected from wild apple forests and nursery of Gongliu County, where *A. mali *were abundant. Ten 50 cm branches with a diameter 1–5 cm were excised from the tree. Branches were randomly selected from the tree with no emergence holes. Next, branches were debarked by knife and chisel to observe larval infestation. Final‐stage larval instars were easily found in late April and the beginning of May. In late June and the beginning of the July, exit holes were observed. To observe adult emergence, all new D‐shaped exit holes were counted in branches. Overall, insect larvae and adult beetles were described following Chamorro et al. (2012). Briefly, 50‐cm excised branches were observed for larval infestation, pupation, and D‐shaped holes. All count data from trees observed in different locations were combined and generated for an average value. Counting period was March to September and acquired data were used to determine borders of each developmental stage in life cycle.

### 2.5 Statistical analysis

StatView software packages were used to perform ANOVA analysis (SAS Institute Inc., Cary, NC, USA).

## 3 RESULTS

### 3.1 Analysis of the mitochondrial *COI* sequences of *A. mali* and hypothesized phylogenetic relationship

**Figure 2:**
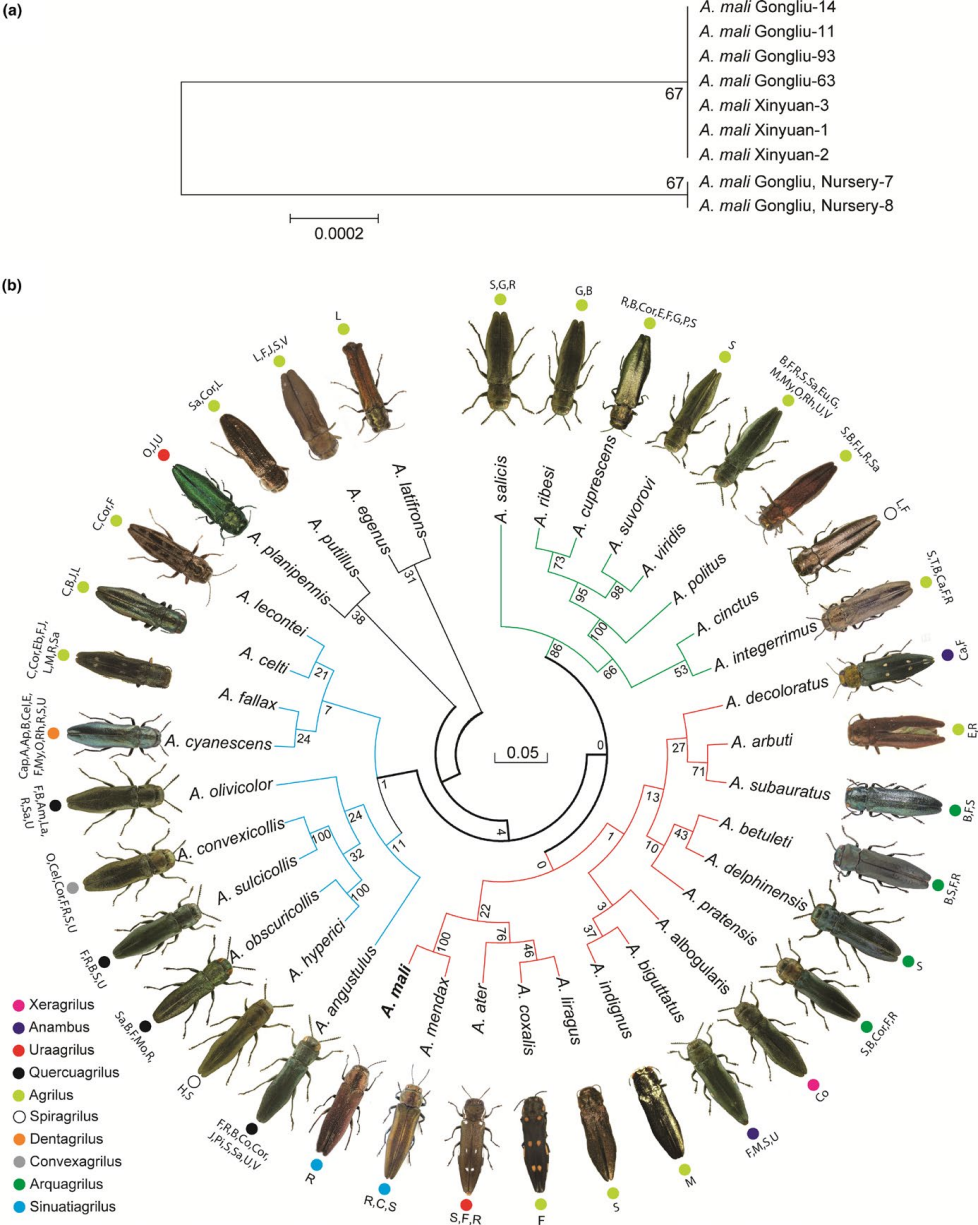
The neighbor‐joining (NJ) tree showing sequence divergences of *COI* for collected specimens (a) and molecular phylogenetic analysis by maximum likelihood method among the *Agrilus *taxa (b). The bootstrap consensus tree inferred from 1,000 replicates is used to represent the evolutionary history of the taxa analyzed. Branches corresponding to partitions reproduced in <50% of bootstrap replicates are collapsed. The percentage of replicate trees in which the associated taxa clustered together in the bootstrap test is shown next to the branches. The evolutionary distances were computed using the Tajima‐Nei method and are in the units of the number of base substitutions per site (a). The evolutionary history was inferred by using the maximum likelihood method based on the Hasegawa‐Kishino‐Yano model. Initial tree(s) for the heuristic search were obtained automatically by applying NJ and BioNJ algorithms to a matrix of pairwise distances estimated using the Maximum Composite Likelihood (MCL). A discrete Gamma distribution was used to model evolutionary rate differences among sites (five categories [+G, parameter = 1.0300]). The rate variation model allowed for some sites to be evolutionarily invariable ([+I], 27.3788% sites). The analysis involved 36 nucleotide sequences. All positions containing gaps and missing data were eliminated. Single‐nucleotide substitution (C to T) is marked as “Y” in the *Agrilus mali* *COI* sequence, which was used in phylogenetic analysis. Colored circle indicate subgenus. Subgeneric classification was obtained from online databases (www.insecta.pro), Database of the Coleopterists Society (www.coleopsoc.org), Wikispecies (www.species.wikimedia.org). Letters indicate host–plants for *Agrilus* species (A, Adoxace; Am, Amaranthaceae; Ap, Apiaceaeae; B, Betulaceae; Ca, Cannabaceae; Cap, Caprifoliaceae; Cel, Celastraceae; Cor, Cornaceae; C, Cannabaceae; Co, Compositae; E, Ericacea; Eb, Ebenaceaee; Eu, Euphorbiaceae; F, Fagaceae; G, Grossulariaceae; H, Hypericaceae; J, Juglandaceae; L, Leguminosae; La, Lamiaceae; M, Malvaceae; My, Myricaceae; Mo, Moraceae; O, Oleaceae; P, Polemoniaceae; Pi, Pinaceae; R, Rosaceae; Rh, Rhamnaceae; S, Salicaceae; Sa, Sapindacea; T, Thymelaeaceae; V, Vitaceaee; U, Ulmaceae)

Multiple alignment analysis revealed that one nucleotide substitution (C/T) was found at position 27 of *COI* sequences among the specimens (Figure 2a). The comparison of the 530 bp aligned *A. mali* *COI *contig sequence with other congeneric species is shown in Supporting Information Table S1. Among the *Agrilus* *COI* orthologs were an average of 290 conserved sites, 240 variable sites, 214 parsimony‐informative sites and 26 singleton sites. Overall, sequence divergence among the congeneric *Agrilus* species following the Tajima‐Nei method was 0.223. The lowest genetic distance was 0.083 with *Agrilus mendax*, which shared 489 conserved sequences with the *A. mali* *COI* sequence, whereas the highest distance was with *A. subauratus*, sharing 412 conserved sites. Nucleotide frequency of the three codon positions in the congeneric species is shown in Supporting Information Table S2. No deletions or insertions were found in *COI* sequences, and additionally, no stop codon was detected when *COI* sequences were translated into amino acids using the invertebrate mitochondrial genetic code. The single‐nucleotide difference found among *COI* of *A. mali* specimens did not influence the amino acid sequence. The 530 bp fragment of the *COI* gene corresponded to 177 codons. The estimate of average evolutionary divergence over all amino acid sequences of congenerics was 0.041, which was less than the average score for nucleotide sequences. Among the *Agrilus* COI amino acid sequences were an average of 136 conserved sites, 33 variable sites, 12 parsimony‐informative sites, and 21 singleton sites.

For the phylogenetic analysis, 35 sequences of *Agrilus* genus were retrieved from databases. Maximum Likelihood (ML) and Bayesian analysis (BA) formed monophyletic groups (Figure 2b; Supporting Information Figure S1). ML and BA demonstrate three groups with outgroups, whereas NJ tree shows four groups with one outgroup. Wood borer, *A. mali* cluster together with *A. mendax *Mannerheim*, A. ater* L.*, A. coxalis *Schaeffer*, A. liragus *Barter & Brown*, A. indignus *Fairmaire,* A. biguttatus *Fabricius*, A. albogularis *Alexeev*, A. pratensis *Ratzeburg,* A. delphinensis* Illiger, *A. betuleti *Ratzeburg,* A. subauratus *Obenberger,* A. arbuti *L.*, *and *A. decoloratus *Kerremans (highlighted in red) (Figure 2b; Supporting Information Figure S1). However, compared to ML and BA, NJ tree demonstrate absence of *A. albogularis* in the group. All trees demonstrated grouping of *A. salicis *Frivaldszky*, A. ribesi *Schaefer, *A. cuprescens *Ménétriés, *A. suvorovi *Obenberger*, A. viridis *Linné*, A. politus *Say*, A. cinctus *Olivier*, *and *A. integerrimus* Ratz. together in single group (highlighted in green) and *A. angustulus *Illiger, *A. hyperici *Creutzer, *A. obscuricollis *Kiesenwetter*, A. sulcicollis *Lacordaire*, A. convexicollis *Redtenbacher*, *and *A. olivicolor *Kiesenwetter*, A. lecontei *Saunders*, A. celti *Knull*, A. fallax *Say*, *and *A. cyanescens. *Kiesenwetter as separate group (highlighted in blue) (Figure 2b; Supporting Information Figure S1). Outgroups in ML and BA clustered in one group except *A. albogularis* in NJ tree. Uncorrected pairwise sequence divergence ranges from 0.02% to 0.36% between species with overall mean distance 0.24. Sequence divergence pairwise comparison within the groups was 0.23 for red group, 0.15 for green group and 0.21 for blue group. ML and BA trees indicate that *A. mali* cluster together with *A. mendax *as a sister group in subgroup formed by other *A. ater, A. coxalis, *and *A. liragus *species showing close relationships*.* Moreover, genetic distances between them remarkably lower compared to other species in the red group. Mean distance between groups was marginal with average 0.25 (blue/red‐0.24, blue/green‐0.25, red/green‐0.26).

Furthermore, the NJ tree developed using amino acid sequences demonstrated close relationships among *A. mali,* *A. mendax*, *A. albogularis*, *A. delphinensis*, *A. celti*, and *A. fallax*, which formed a cluster but visually differed from one another (Supporting Information Figure S2). In general, the divergence of COI sequences among congeneric species is over 2% (Hebert, Ratnasingham, & deWaard, 2003), which is consistent with our results of an overall distance among the *Agrilus* species of 0.223, whereas the average distance of *A. mali* with other congeneric species was 0.226.

### 3.2 Insect description

**Figure 3:**
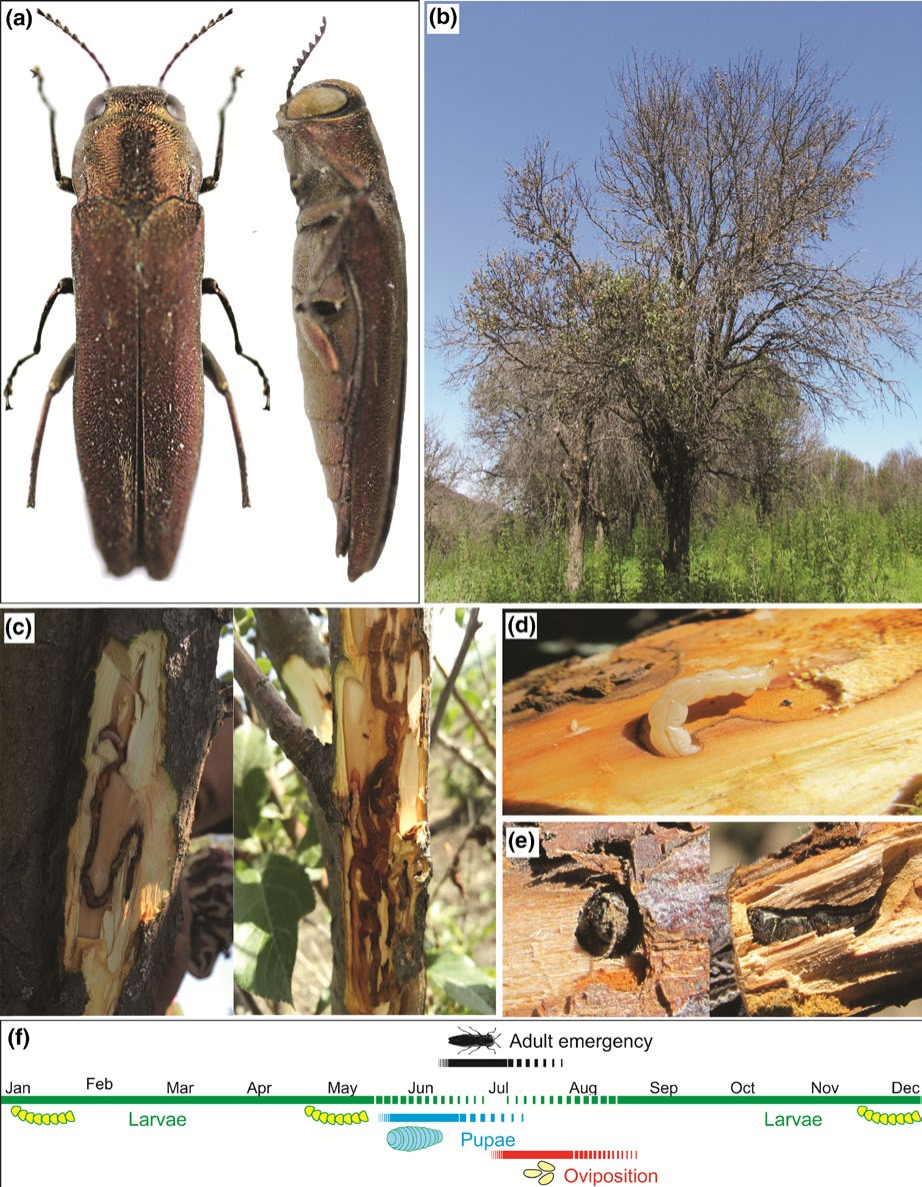
*Agrilus mali* infestations of wild apple and beetle behaviors. Dorsal and lateral views of *A. mali *morphology (a); wild apple tree heavily infested with *A. mali* (b); typical serpentine galleries caused by larvae (c); mature larva in the young stem (d); adult in the stem and D‐shaped adult emergence hole (e). Summary of the life cycle of *A. mali* in the wild apple forests of Tianshan Mountains (f)

Adults are medium‐sized insects 7–10 mm long that are variable in color (dark brown or dark bronze) and shiny (metallic) (Figure 3a). Usually ventral side unicolored with greenish shade. Body shape cuneiform or parallel; head usually impressed medially; predominant sculpture of vertex is rugae irregular, dense. Eyes are small or moderate, protruding from head outline, lower margin of eye below antennal socket. Antennae are small, slender shaped. Pronotum transverse shaped, maximal width at middle lateral margin; anterior margin is narrower than posterior angle. Anterior lobe moderate with arcuate shape at level with anterior pronotal angles. Apex of posterior angles is sharp with obtuse shape. Scutellum size is rudimentary with absolute carina; disk with carinal prehumerus. Elytra unicolored or with elytral apices dark carmine; humeral carina absent; apices wide, separately arcuate; elytral pubescence distal only. Prosternal process narrowed flat, sides straight. Lengths of all legs are the same. Larvae are 18–20 mm long, off‐white in color, and with 12 segments. Larval abdominal segments are generally oval. Eggs are oblong, and newly deposited eggs are initially creamy white then gradually yellow over time 10–14 days before hatching.

### 3.3 Insect life cycle

**Figure 4:**
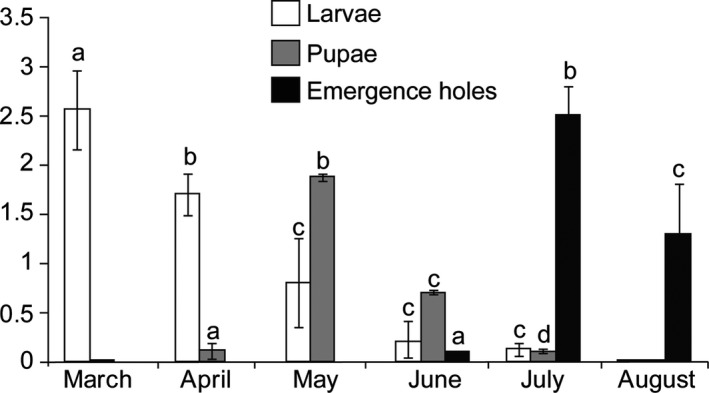
Average number of observed larvae, pupa and emergence holes in wild apple forests. Ten 50 cm branches per tree were observed for larvae, pupae and emergence holes. Data from each randomly chosen 10 trees combined together. Shown values are mean (± *SE*) of 100. Different letters show significant differences among months of each stage as determined by one‐way ANOVA, followed by a Fisher PLSD post hoc test (*p* ≤ 0.05)

An expedition was conducted to the Ili‐Kazakh Autonomous Prefecture of the Xinjiang‐Uyghur Autonomous Region to survey the mortality in wild apple forests caused by the invasive insect *A. mali*. We observed that the damage to wild apple trees caused by the widely distributed *A. mali* in Xinyuan County of Ili was much more serious than that in Gongliu County. Based on monthly regular inspection in Gongliu and Xinyuan counties of the Tianshan Mountains, we determined the life cycle of *A. mali*. In late July until the beginning of September, females of *A. mali* laid eggs on the surface of apple tree stems on injured or diseased bark or in crevices in branches from which hatched larvae bored into the stem to reach the xylem portion of the branches. Usually, larvae can be found more in young branches compared to trunk (Dr. Lu Zhaozhi, unpublished data). Damaged branches of infested apple (Figure 3b) formed inward bulging that resulted in the bark squeezed between the stem and the crack. Phloem, cambium, and outer xylem tissues were fed on by larvae that formed serpentine galleries (Figure 3c,d) to the end of June, before entering the woody portion to build pupation chambers. The bark of the infested areas blackened and dried. Depending on environmental conditions and stem age, pupation occurred over 2–3 months beginning from late April to the end of July (Figure 3f). Adult emergence occurred in the beginning of June and continued to the end of July (Figure 3e,f). In addition to the regular inspection of developmental stages, beginning and end of the number of infestation, pupation and emergence rates were determined to start from March. Average numbers of insect developmental stages were determined in excised infested branches in 50 cm long with diameter 1–5 cm per 10 trees in the forests and nursery of Gongliu County. Results revealed that the number of larvae in March was 2.56 ± 0.4 per 50 cm branch but decreased from the beginning of April which showed 1.7 ± 0.9 and very few of them were found in June and July with 0.2 ± 0.09 and 0.12 ± 0.07 larvae, respectively (Figure 4). The decreased number of larvae correlated with increased rate of pupation in May and in June that estimated 1.87 ± 0.04 and 0.7 ± 0.02 pupae per 50 cm branch but decreased in late June and the beginning of July. The rate of larval infestation and pupation in these months can provide boundary of life cycles (Figure 4). The high emergency rate was observed in July and August. However, in the late summer session, few larvae still could be found in apple branches which were not fully developed or transitioned to the second overwintering period (Dr. Lu Zhaozhi, unpublished data). Furthermore, observation in two different locations varied in infestation rates. A large area of wild apple forest was heavily damaged in Xinyuan counties compared to Gongliu (Figure 1).

## 4 DISCUSSION

Molecular phylogeny provides insight into close level relationship in the genus *Agrilus*. In this molecular analysis, we explored the relationship of *A. mali* with other congeneric species. Since first reported in 1924 (Matsumura, 1924), detailed morphological traits and its phylogenetic status of *A. mali* have not been fully studied. The identical *COI* sequences from specimens collected in Gongliu and Xinyuan counties indicated that individuals were all one species, although one nucleotide differences were detected with individuals collected from the apple nursery in Gongliu. Genetic variation among *Agrilus* species in all ML groups was marginal. Clustering network analysis revealed that *A. mali* most closely grouped with *A. mendax *and was less related to *A. ater, A. coxalis*, and *A. liragus. *Generally, ML tree was consistent to morphologically classified species by subgenera. For example, subgenera *Quercuagrilus*, *Agrilus*, and *Arquagrilus*, clustered separately (Fisher, 1928).

Recent work by Kelnarova, Jendek, Grebennikov, and Bocak (2018) reported the first molecular phylogeny of *Agrlius* genera using three DNA barcodes. Even though the three mitochondrial markers based phylogeny covers only ~3% of the known *Agrilus* diversity it represents the most extensive dataset built for DNA‐delimited species identification within this genus so far. Marker *COI1* used in our study corresponds to the *cox1–5′ *markers which fully covered by other *cox1–3′* marker (Kelnarova et al., 2018). Despite single marker, our phylogenetic has a similarity with reported recent work. For example, *A. salicis* and *A. sulcicollis* group clusters are almost same, although in both studies many species were missed in each tree. Here, we attempted to show a hypothesized phylogenetic status of *A. mali* among other *Agrilus* species. Although using only one genetic marker is inconsistent and might not support clear phylogenetic status of species including haplotype diversity (Kelnarova et al., 2018). However, *COI* barcode and phylogenetic clustering with other congeneric species (Kelnarova et al., 2018) are enough to make a preliminary conclusion.

Thirty‐two host–plant species are food sources for *Agrilus* species (Jendek & Poláková, 2014) which are distributed differently among groups. Interestingly, group highlighted in red attack narrower range of host–plant species compared to other groups in the hypothesized tree. Both *A. mali* and *A. mendax* are specialists that attack Rosaceae species, *Malus* and *Sorbus*, respectively (Jendek & Poláková, 2014). Understanding the diversification processes is one of the main question in evolutionary biology. Insect attacking diverse host–plants besides main host provides opportunity for adaptive speciation. Insect in their native habitat can co‐evolve together with local host–plants. However, other species of same host genus can be more sensitive because of lack of co‐evolution. For example, North American *Fraxinus* spp. is more sensitive to EAB than Asian species. Similarly, *A. mali *lead to extensive mortality of wild apple than other *Malus* species in native habitat of east China.

Understanding the life cycle of beetles allows using contact or systemic insecticides to treat plants in order to improve management. In addition, molecularly identified agricultural pests or invasive insects can provide a prerequisite to choose appropriate natural enemies in order to time releases during larvae stages or before emergency. The effectiveness of molecular methods has been referred to “who eats whom” questions in food‐web ecology (Furlong, 2015; Hanner, Lima, & Floyd, 2009). The life cycle in Tianshan Mountains takes 1 year but could be extended for a second year depending on weather or late oviposition period (Dr. Lu Zhaozhi, Personal communication). *Agrilus mali* rarely attacks trunks but lays eggs on surfaces of young branches or new shoots. This might be due to a thinner layer of bark in branches compared to old trunk. Oviposition starts in July and after 1–2 weeks, newly hatched eggs bore into the phloem and start constructing larval galleries. However, galleries have less serpentine path process compared to galleries of infamous *A. planipennis *which might be due to smaller diameters of branches. Larval feeding behavior in the branch, particularly the formation of serpentine galleries, could interrupt upward nutrient movement to branches, resulting in dryness of the entire or partial trunk depending on the damaged area of the branch.

Overwintering period starts for larvae with decreasing temperature in September and continues through late March in Tianshan Mountains. During this period, larval instars did not grow much considerably but all larval instars could be found. However, adult insect overwintering were not observed and was not reported in literature. Climate of Tianshan Mountains is strongly continental based on Koppen climate classification (Peel & McMahon, 2007) with very cold winter and hot summer (Chen et al., 2013). Most wood borers have ability to survive cold during overwintering period. Glycerol accumulated in larvae serves as a major cryoprotectant during the winter (Crosthwaite, Sobek, Lyons, Bernards, & Sinclair, 2011; Li, Shi, Xue, & Mao, 2014). Infestation rate is lower in northern range and more resistance hosts but generally has 2‐year life cycle for EAB whereas below northern range, it is typically 1 year (Orlova‐Bienkowskaja & Bienkowski, 2016). Larval growth starts from the middle of March depending on weather. In the middle of May, approximately all of larvae were third and fourth instar. Larvae could also be found in the middle of the summer that passed to the second year life cycle that may belong to the previous cohort. Pupation and emergence period also depend on weather that varies between 1 and 2 months beginning of June. Comparison of life cycles of EAB in different location in China, Russia and USA showed that life history varied according to weather conditions, primarily the duration of the warm period (Orlova‐Bienkowskaja & Bienkowski, 2016). For example, latitude and host condition influence on the development of *A. anxius* Gory (Beer, 1949) and the lower temperature in *Agrilus biguttatus* Fabricus development (Reed, Denman, Leather, Forster, & Inward, 2018). Likely, differences in timing of stages development influenced by timing of adult emergency, frequencies of adult mating, fecundity of females, and oviposition period (Marshall, Miller, Lelito, & Storer, 2013; Orlova‐Bienkowskaja & Bienkowski, 2016). Furthermore, timing of development could also be affected by condition of host. Survey of infested tree demonstrated that adults lay eggs on younger twigs rather than old trunk and/or bigger branch. Probably, newly hatched neonates can easily bore into the thinner bark of younger twigs. Additionally, incomplete developed larvae of 1‐year cycle might pass to second year that might be related to host condition and density of larval population (Orlova‐Bienkowskaja & Bienkowski, 2016). Average life of adult beetles that generally feed on leaves was 45 days. Timing of developmental stages of *A. mali* can provide significant information for the pesticide treatment.

The recent invasion of *A. mali* has caused more than 40% of apple forests to die (Wang, 2013). The consequence of the insect invasion can be clearly observed in the forests of Xinyuan County that led to huge mortality of wild apple population. In recent years, the invasion has expanded to other neighboring counties such as Gongliu and Yining (Figure 1b). Despite government efforts to prevent insect spread by pesticide application via air‐spray or felling of trees, insects still escape to other locations (Liu, Zhang, Yue, & Wen, 2016). Our field investigations during the last 2 years showed limited numbers of larvae or adult beetles in Xinyuan apple populations that likely migrated to other places because of high tree mortality. Xinyuan could serve as the center for insect distribution to other locations like Gongliu. Apparently, adult beetles cannot fly far, and therefore distribution is not rapid (Sun, Liang, & Sun, 1979). However, human intervention might accelerate the expansion of insects by seedling transportation or grafting practices (Dr. Lu Zhaozhi, personal communication).

Currently, wild apple populations in Gongliu suffer from insect attacks which might lead to an extensive decrease in apple forests. Moreover, *A. mali* invasion might not only restricted to wild apple forest of western China but could be also passed into western Tianshan mountains area, including Kazakhstan (Figure 1). During our expedition to the central Asian part of Tianshan, a field investigation was conducted in several *M. sieversii* populations of Ili‐Altao, Jugar‐Altao, and Chimkent (Kazakhstan) in 2017. We found that the apple populations had been seriously damaged and extensive tree mortality was observed (Figure 1c). However, Kazakh scientists and local gardeners did not establish causal agent of tree mortality. The wild apple forest in Central Asian Tianshan part could be potential area under high risk of insect invasion. Therefore, *A. mali *is an important pest that could be considered as an international threat. Development of biological or chemical pest management programs for the whole Tianshan is challenging and requires developing action programs to stop insect distribution along with other central Asian countries.

In this study, we describe an invasive *A. mali *and its taxonomic status, including the relationship with other *Agrilus* species, DNA barcodes, and the life cycle in western China that will promote further research and assist the development of biological control. Furthermore, based on field investigations, we considered that wild apple forest of Xinyuan as the center for *A. mali* invasion to other locations in western China which occurred for the last 20 years. International cooperation is imminent to jointly develop a program to prevent *A. mali *expansion to other potential distribution area along Tianshan Mountains.

## CONFLICT OF INTEREST

None declared.

## AUTHOR CONTRIBUTION

TAB performed the experimental work. TAB and DZ participated in the design of the study. TAB and ZL described insects. TAB, XL, and DZ collected insect larvae. TAB, ZL, and DZ studied life cycle in field. TAB, XL, and DZ analyzed the data. TAB and DZ conceived of the study and edited the manuscript. TAB drafted the manuscript. DZ provided all of the fundings.

## Data Availability

*COI* sequences in available in NCBI. GenBank accessions KY982844, KY982844, KY982846, KY982847, KY982848, KY982849, KY982850, KY982851, KY982852.
