# Supplementary figures and images for: Agrilus mali Matsumara (Coleoptera: Buprestidae), a new invasive pest of wild apple in western China: DNA barcoding and life cycle
Source: Ecol Evol. 2018 Dec 27;9(3):1160–72. doi: 10.1002/ece3.4804 (PMC6374668; doi:10.1002/ece3.4804)

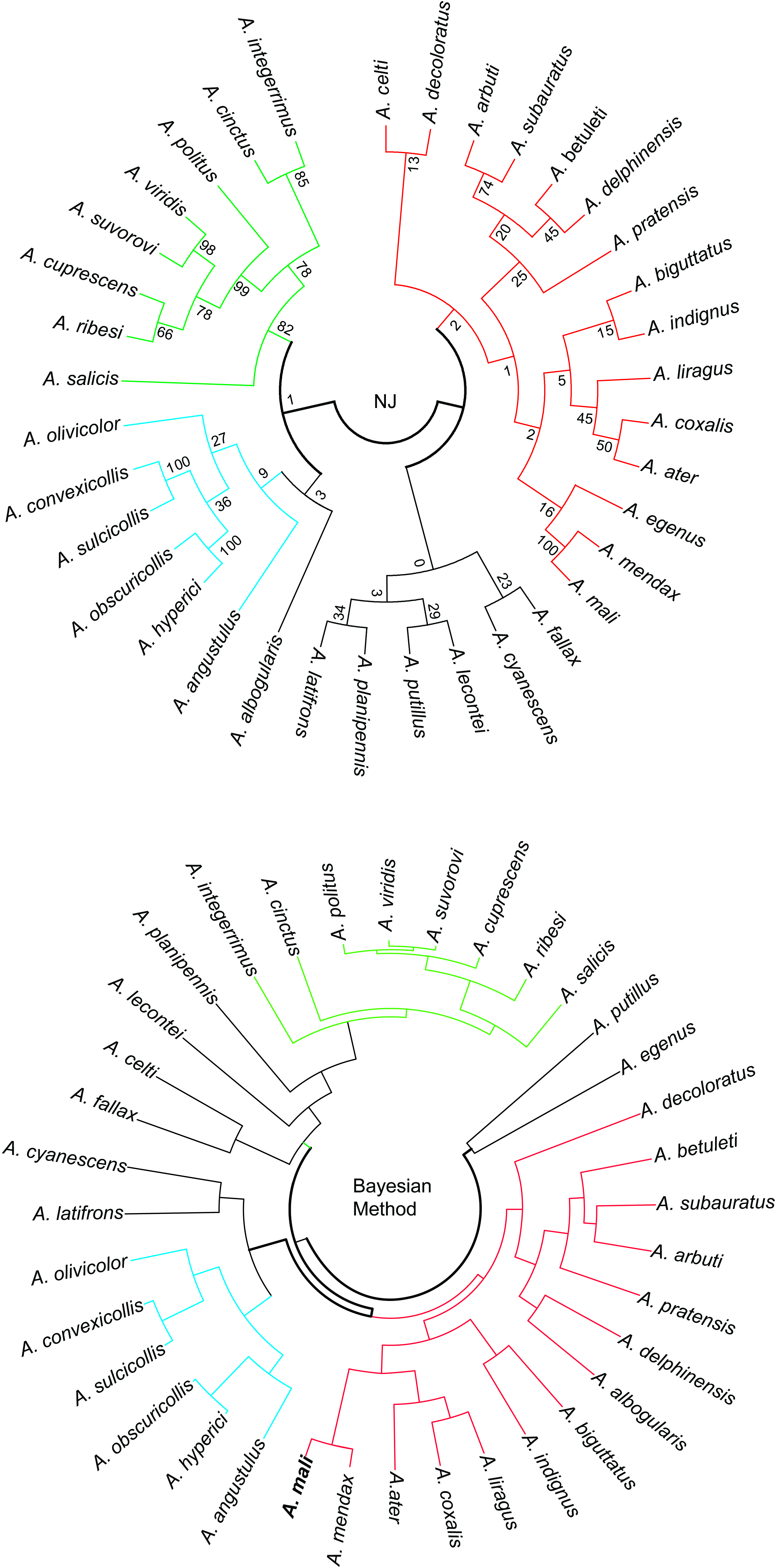

Supplement: Supplementary file 1 [file ECE3-9-1160-s001.tif]

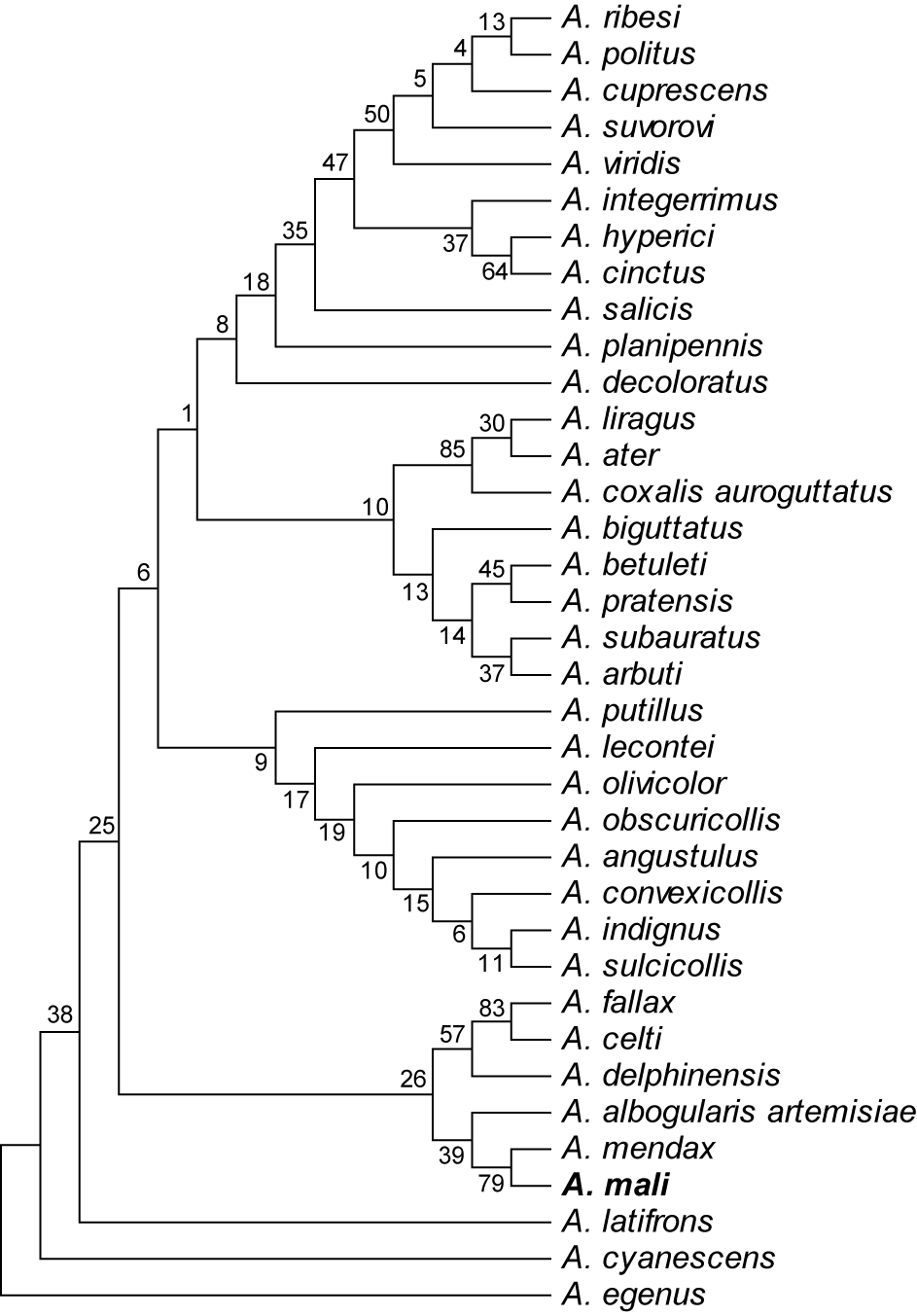

Supplement: Supplementary file 2 [file ECE3-9-1160-s002.tif]
