# Supplementary material for: Agrilus mali Matsumara (Coleoptera: Buprestidae), a new invasive pest of wild apple in western China: DNA barcoding and life cycle
Source: Ecol Evol. 2018 Dec 27;9(3):1160–72. doi: 10.1002/ece3.4804 (PMC6374668; doi:10.1002/ece3.4804)
Supplement: Supplementary file 3 [file ECE3-9-1160-s003.docx]

**Table S1.** Genetic analysis of partial *A. mali* *COI* sequence with other cogeneric *Agrilus* species

| **Species** | **Sequence similarity** | **Genetic distance** | **St. error** | **Conserved sites** | **Variable sites** | **GeneBank Acc. No.** |
| --- | --- | --- | --- | --- | --- | --- |
| *A. mendax* | 92 | 0.083 | 0.013 | 489 | 41 | KJ962013 |
| *A. coxalis* | 84 | 0.185 | 0.021 | 444 | 86 | JF719861 |
| *A. convexicollis* | 83 | 0.189 | 0.021 | 442 | 88 | KM446658 |
| *A. pratensis* | 83 | 0.191 | 0.021 | 441 | 89 | KM452415 |
| *A. sulcicollis* | 83 | 0.195 | 0.022 | 440 | 90 | KT250507 |
| *A. olivicolor* | 83 | 0.198 | 0.022 | 439 | 91 | KM450616 |
| *A. liragus* | 83 | 0.204 | 0.022 | 436 | 94 | KM848158 |
| *A. fallax* | 82 | 0.203 | 0.022 | 436 | 94 | KJ087651 |
| *A. lecontei* | 82 | 0.215 | 0.023 | 432 | 98 | BBCCM681 |
| *A. delphinensis* | 82 | 0.207 | 0.023 | 435 | 95 | KM445306 |
| *A. politus* | 82 | 0.207 | 0.022 | 435 | 95 | KM845661 |
| *A. ater* | 82 | 0.209 | 0.023 | 434 | 96 | FBCOB921 |
| *A. cyanescens* | 82 | 0.209 | 0.023 | 434 | 96 | KM441105 |
| *A. viridis* | 82 | 0.210 | 0.023 | 434 | 96 | HM909145 |
| *A. planipennis* | 82 | 0.213 | 0.023 | 432 | 98 | KM845113 |
| *A. albogularis* | 81 | 0.218 | 0.023 | 431 | 99 | KM439556 |
| *A. integerrimus* | 81 | 0.219 | 0.023 | 430 | 100 | KM451147 |
| *A. ribesi* | 81 | 0.222 | 0.024 | 430 | 100 | KT250501 |
| *A. cinctus* | 81 | 0.223 | 0.024 | 429 | 101 | KM451807 |
| *A. biguttatus* | 81 | 0.224 | 0.024 | 429 | 101 | KM286083 |
| *A. cuprescens* | 81 | 0.224 | 0.024 | 429 | 101 | KT250506 |
| *A. betuleti* | 81 | 0.225 | 0.024 | 428 | 102 | HM909146 |
| *A. egenus* | 81 | 0.242 | 0.025 | 421 | 109 | INRMA1872 |
| *A. decoloratus* | 80 | 0.230 | 0.024 | 426 | 104 | KM364310 |
| *A. salicis* | 80 | 0.236 | 0.024 | 424 | 106 | KM451582 |
| *A. obscuricollis* | 80 | 0.240 | 0.025 | 423 | 107 | KM439788 |
| *A. hyperici* | 80 | 0.240 | 0.025 | 423 | 107 | KM447291 |
| *A. angustulus* | 80 | 0.243 | 0.025 | 422 | 108 | KJ967113 |
| *A. arbuti* | 80 | 0.247 | 0.025 | 420 | 110 | KM364308 |
| *A. latifrons* | 79 | 0.252 | 0.026 | 418 | 112 | KM364420 |
| *A. subauratus* | 79 | 0.268 | 0.027 | 412 | 118 | KJ768194 |

*These COI sequences were obtained from DNA barcode database, the Barcode of Life Data Systems BOLD Systems (http://www.boldsystems.org).
