# Supplementary material for: Agrilus mali Matsumara (Coleoptera: Buprestidae), a new invasive pest of wild apple in western China: DNA barcoding and life cycle
Source: Ecol Evol. 2018 Dec 27;9(3):1160–72. doi: 10.1002/ece3.4804 (PMC6374668; doi:10.1002/ece3.4804)
Supplement: Supplementary file 4 [file ECE3-9-1160-s004.docx]

**Table S2** Nucleotide frequencies (thymine (T), cytosine (C), adenine (A) and guanine (G) of the three codon positions in different *Agrilus* species

| **Species** | **T-1** | **C-1** | **A-1** | **G-1** | **T-2** | **C-2** | **A-2** | **G-2** | **T-3** | **C-3** | **A-3** | **G-3** |
| --- | --- | --- | --- | --- | --- | --- | --- | --- | --- | --- | --- | --- |
| *A. lecontei* | 32.3 | 20.9 | 28.5 | 18.3 | 32.3 | 21.0 | 28.5 | 18.1 | 32.4 | 21.0 | 28.6 | 18.0 |
| *A. ater* | 36.2 | 19.8 | 27.7 | 16.2 | 36.3 | 19.8 | 27.8 | 16.1 | 36.4 | 19.9 | 27.8 | 15.9 |
| *A. viridis* | 34.3 | 19.6 | 28.1 | 17.9 | 34.4 | 19.7 | 28.2 | 17.8 | 34.5 | 19.7 | 28.2 | 17.6 |
| *A. betuleti* | 32.3 | 20.9 | 29.1 | 17.7 | 32.3 | 21.0 | 29.1 | 17.6 | 32.4 | 21.0 | 29.2 | 17.4 |
| *A. egenus* | 37.9 | 16.6 | 29.2 | 16.2 | 38.0 | 16.6 | 29.3 | 16.1 | 38.1 | 16.7 | 29.4 | 15.9 |
| *A. coxalis* | 34.2 | 20.8 | 28.7 | 16.4 | 34.2 | 20.8 | 28.7 | 16.3 | 34.3 | 20.8 | 28.8 | 16.1 |
| *A. fallax* | 32.1 | 21.7 | 30.0 | 16.2 | 32.1 | 21.7 | 30.1 | 16.1 | 32.2 | 21.8 | 30.1 | 15.9 |
| *A. subauratus* | 31.5 | 21.5 | 28.7 | 18.3 | 31.6 | 21.6 | 28.7 | 18.1 | 31.6 | 21.6 | 28.8 | 18.0 |
| *A. angustulus* | 31.1 | 20.9 | 30.9 | 17.0 | 31.2 | 21.0 | 31.0 | 16.8 | 31.3 | 21.0 | 31.1 | 16.7 |
| *A. biguttatus* | 34.0 | 19.4 | 28.1 | 18.5 | 34.0 | 19.5 | 28.2 | 18.3 | 34.1 | 19.5 | 28.2 | 18.2 |
| *A. arbuti* | 31.3 | 22.6 | 27.5 | 18.5 | 31.4 | 22.7 | 27.6 | 18.3 | 31.4 | 22.7 | 27.7 | 18.2 |
| *A. decoloratus* | 31.3 | 22.1 | 29.1 | 17.5 | 31.4 | 22.1 | 29.1 | 17.4 | 31.4 | 22.2 | 29.2 | 17.2 |
| *A. latifrons* | 32.6 | 21.1 | 27.7 | 18.5 | 32.7 | 21.2 | 27.8 | 18.3 | 32.8 | 21.2 | 27.8 | 18.2 |
| *A. albogularis* | 34.5 | 20.9 | 27.4 | 17.2 | 34.6 | 21.0 | 27.4 | 17.0 | 34.7 | 21.0 | 27.5 | 16.9 |
| *A. obscuricollis* | 30.8 | 22.5 | 27.9 | 18.9 | 30.8 | 22.5 | 28.0 | 18.7 | 30.9 | 22.5 | 28.0 | 18.6 |
| *A. cyanescens* | 30.6 | 22.3 | 29.2 | 17.9 | 30.6 | 22.3 | 29.3 | 17.8 | 30.7 | 22.3 | 29.4 | 17.6 |
| *A. delphinensis* | 32.3 | 22.3 | 28.3 | 17.2 | 32.3 | 22.3 | 28.4 | 17.0 | 32.4 | 22.3 | 28.4 | 16.9 |
| *A. convexicollis* | 33.0 | 20.2 | 30.0 | 16.8 | 33.1 | 20.2 | 30.1 | 16.6 | 33.1 | 20.3 | 30.1 | 16.5 |
| *A. hyperici* | 30.8 | 22.5 | 27.9 | 18.9 | 30.8 | 22.5 | 28.0 | 18.7 | 30.9 | 22.5 | 28.0 | 18.6 |
| *A. olivicolor* | 32.6 | 20.2 | 29.6 | 17.5 | 32.7 | 20.2 | 29.7 | 17.4 | 32.8 | 20.3 | 29.7 | 17.2 |
| *A. integerrimus* | 35.1 | 20.4 | 26.8 | 17.7 | 35.2 | 20.4 | 26.8 | 17.6 | 35.2 | 20.5 | 26.9 | 17.4 |
| *A. salicis* | 32.6 | 21.7 | 29.4 | 16.2 | 32.7 | 21.7 | 29.5 | 16.1 | 32.8 | 21.8 | 29.5 | 15.9 |
| *A. cinctus* | 32.3 | 21.7 | 26.8 | 19.2 | 32.3 | 21.7 | 26.8 | 19.1 | 32.4 | 21.8 | 26.9 | 18.9 |
| *A. pratensis* | 34.3 | 19.2 | 29.4 | 17.0 | 34.4 | 19.3 | 29.5 | 16.8 | 34.5 | 19.3 | 29.5 | 16.7 |
| *A. planipennis* | 33.8 | 19.4 | 29.6 | 17.2 | 33.8 | 19.5 | 29.7 | 17.0 | 33.9 | 19.5 | 29.7 | 16.9 |
| *A. politus* | 34.7 | 19.6 | 27.7 | 17.9 | 34.8 | 19.7 | 27.8 | 17.8 | 34.8 | 19.7 | 27.8 | 17.6 |
| *A. liragus* | 34.5 | 20.8 | 27.5 | 17.2 | 34.6 | 20.8 | 27.6 | 17.0 | 34.7 | 20.8 | 27.7 | 16.9 |
| *A. ribesi* | 33.0 | 20.6 | 28.3 | 18.1 | 33.1 | 20.6 | 28.4 | 18.0 | 33.1 | 20.6 | 28.4 | 17.8 |
| *A. cuprescens* | 33.6 | 20.2 | 29.1 | 17.2 | 33.6 | 20.2 | 29.1 | 17.0 | 33.7 | 20.3 | 29.2 | 16.9 |
| *A. sulcicollis* | 32.3 | 20.9 | 29.4 | 17.4 | 32.3 | 21.0 | 29.5 | 17.2 | 32.4 | 21.0 | 29.5 | 17.0 |
| *A. mali Farmland* | 35.5 | 18.1 | 29.6 | 16.8 | 35.5 | 18.1 | 29.7 | 16.6 | 35.6 | 18.2 | 29.7 | 16.5 |
| *A. mali Gongliu* | 35.3 | 18.3 | 29.6 | 16.8 | 35.3 | 18.3 | 29.7 | 16.6 | 35.4 | 18.4 | 29.7 | 16.5 |
| *A. mendax* | 35.3 | 18.1 | 30.4 | 16.2 | 35.3 | 18.1 | 30.4 | 16.1 | 35.4 | 18.2 | 30.5 | 15.9 |
| Average | 33.3 | 20.5 | 28.7 | 17.5 | 33.3 | 20.6 | 28.8 | 17.3 | 33.4 | 20.6 | 28.8 | 17.2 |
